# Combined anatomical optical coherence tomography and intraluminal pressure reveal viscoelasticity of the *in vivo* airway

**DOI:** 10.1117/1.JBO.23.10.100501

**Published:** 2018-10-22

**Authors:** Santosh Balakrishnan, Ruofei Bu, Nicusor Iftimia, Hillel Price, Carlton Zdanski, Amy L. Oldenburg

**Affiliations:** a University of North Carolina at Chapel Hill, Department of Biomedical Engineering, Chapel Hill, North Carolina, United States; b Physical Sciences Inc., Andover, Massachusetts, United States; c University of North Carolina at Chapel Hill, Department of Physics and Astronomy, Chapel Hill, North Carolina, United States; d University of North Carolina at Chapel Hill, Department of Otolaryngology/Head and Neck Surgery, Chapel Hill, North Carolina, United States; e University of North Carolina at Chapel Hill, Biomedical Research Imaging Center, Chapel Hill, North Carolina, United States

**Keywords:** optical coherence tomography, endoscopic OCT, dynamic airway imaging, elastography

## Abstract

It is hypothesized that the local, viscoelastic (time-dependent) properties of the airway are important to accurately model and ultimately predict dynamic airway collapse in airway obstruction. Toward this end, we present a portable, endoscopic, swept-source anatomical optical coherence tomography (aOCT) system combined with a pressure catheter to capture local airway dynamics *in vivo* during respiration. aOCT scans were performed in the airways of a mechanically ventilated pig under paralysis with dynamic and static ventilation protocols. Validation of dynamic aOCT luminal cross-sectional area (CSA) measurements against Cine CT, obtained during the same exam, showed an aggregate difference of 15%±3%. aOCT-derived CSA obtained in the *in vivo* trachea also exhibited hysteresis as a function of pressure, depicting the viscoelastic nature of the airway wall. The volumetric imaging capabilities were validated by comparing aOCT- and CT-derived geometries of the porcine airway spanning nine generations from the trachea to the bronchioles. The ability to delineate regional differences in airway viscoelastic properties, by measuring airway deformation using aOCT combined with intraluminal pressure, paves the way to patient-specific models of dynamic airway collapse.

We describe the design and validation of a portable, swept-source anatomical optical coherence tomography (aOCT) system for quantitative endoscopy of the airway lumen with synchronous intraluminal pressure measurement that can be used to characterize the viscoelastic properties of the airway. Previous work has demonstrated the utility of OCT in dynamic imaging of the airways,^1^^,^^2^ accurate measurements of airway shape and dimensions,^2^^,^^3^^–^^5^ and estimates of airway elastic properties in airway obstruction.^2^^,^^6^^,^^7^ In studies that used airway pressures, either pressures measured at the patient’s mouth,^1^ nose,^2^ or transmural pressure, calculated as the difference between the pressure at the mouth and the esophagus^6^ were used in conjunction with OCT. We hypothesize that better estimates of local airway elastic properties are enabled by the concomitant use of a pressure-sensing catheter at the site of imaging. A pressure catheter was used by Robertson et al.,^7^ but their work did not explore viscoelastic response of the *in vivo* animal trachea. In this study, we report the use of long-range OCT (aOCT) in conjunction with an intraluminal pressure catheter and explore the time-dependent mechanical properties (viscoelasticity) of the airway using this setup. This expands potential applications of aOCT to include upper airway obstructive disorders, which require long-range imaging and are characterized by dynamic airway collapse,^2^^,^^5^ the latter of which is expected to be influenced by tissue viscoelastic properties. Additionally, the validation of OCT airway imaging has previously been performed against CT scans that were obtained as a part of a different exam^1^^,^^3^^,^^4^^,^^6^ or when performed within the same exam^2^ used one time-point within the respiratory cycle for comparisons; here, we present dynamic imaging studies of the airways that have been validated against Cine CT images obtained within the same animal subject, throughout a controlled respiratory cycle, under identical conditions.

A block diagram of our aOCT and pressure acquisition system is shown in Fig. 1, while additional details are shown in Figs. 2(a) and 2(c), respectively. The OCT system utilizes a vertical-cavity surface-emitting laser (VCSEL) wavelength-swept source (SL1310V1, Thorlabs Inc.; center wavelength ∼1300  nm, sweep range ∼120  nm, and 100-kHz sweep rate) in a fiber-optic Mach–Zehnder interferometer with a variable delay reference arm. The sample arm consists of a fiber-optic catheter with a protective sheath (Ref. 8; ∼0.85-mm outer diameter, ∼175-cm length, and flexible Nitinol driveshaft) connected to a rotation/translation scanner (Physical Sciences Inc.). The resulting interference signal is detected by a balanced photodetector (PDB480C, Thorlabs Inc.) and digitized by a data acquisition (DAQ) card (ATS9360, Alazar Technologies Inc.). The laser also outputs a k-clock, which is used as the sampling clock by the DAQ. The resulting imaging range is ∼12  mm. An antialiasing filter is used to reduce noise from structures beyond the 12-mm imaging range and a 10-dB attenuator is used to prevent saturation from strongly reflecting features. A variable delay box (DB64, Stanford Research Systems) is used to synchronize the k-clock signal with the OCT interferometer signal. The laser outputs ∼23  mW of power and the measured power at the output of the catheter is ∼13  mW. The system has a measured axial resolution of 12.6±2.7  μm over the imaging range. The SNR at 1.8-mm distance is 105.7 dB, reducing to 83.1 dB at 11.8 mm primarily due to divergence of the beam from the catheter.

**Fig. 1:**
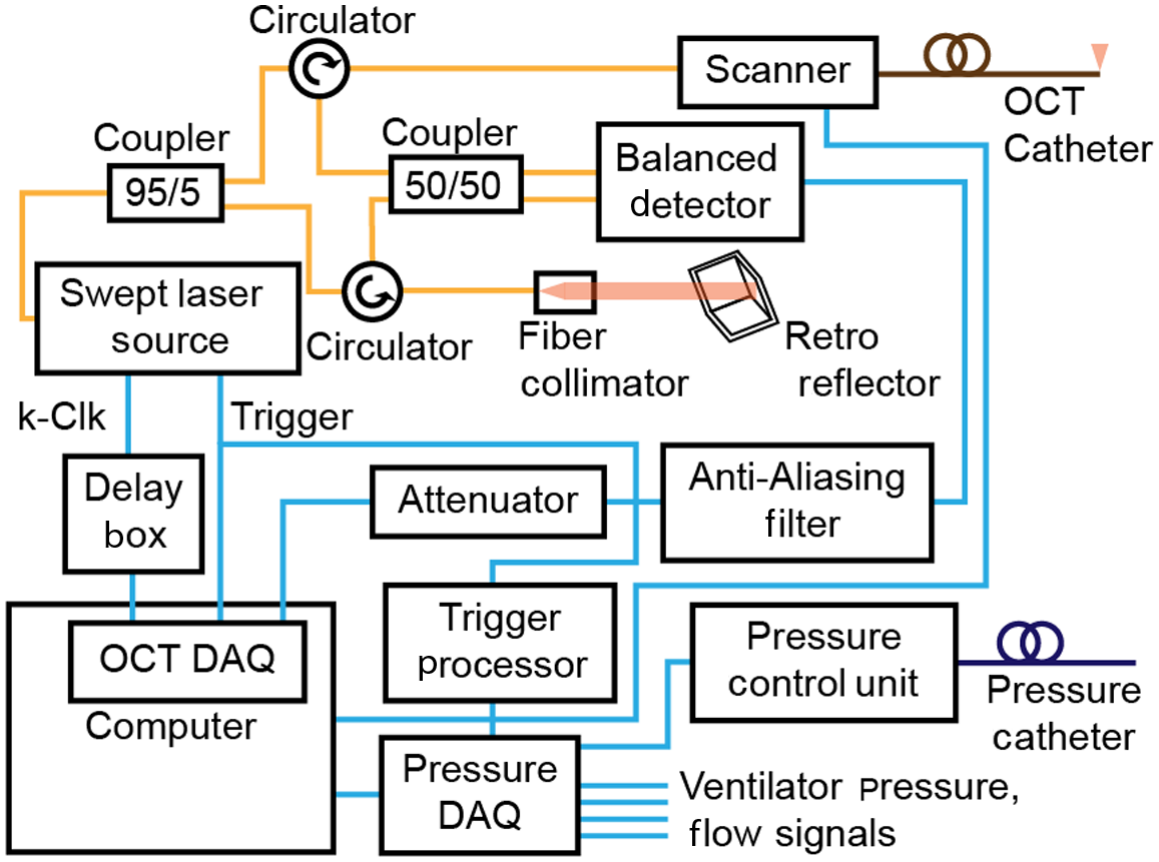
System block diagram; blue and yellow lines indicate electrical and optical connections, respectively.

**Fig. 2:**
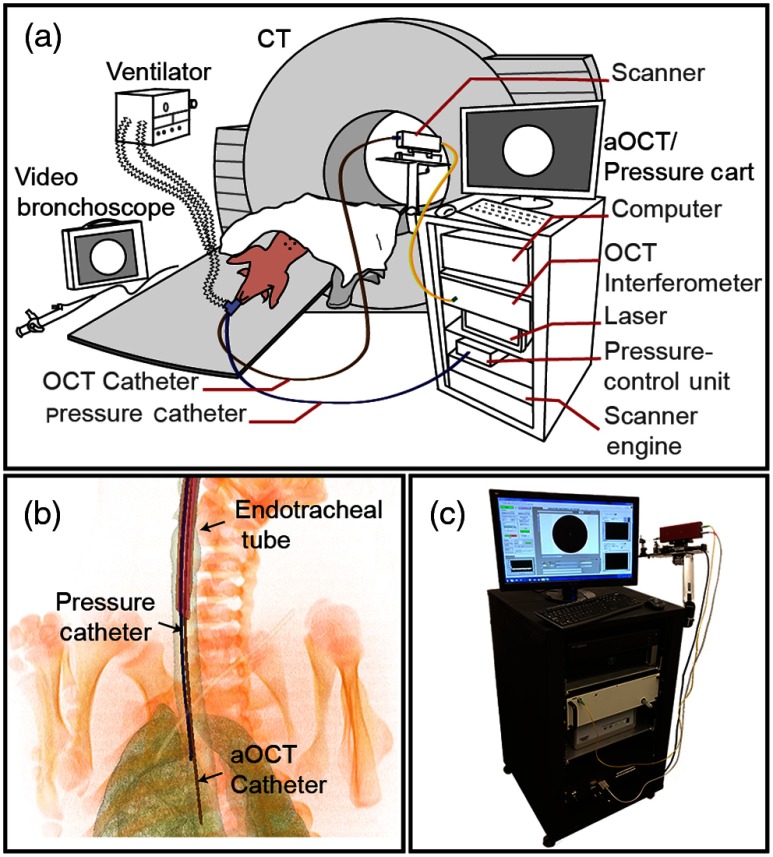
(a) Setup used for *in vivo* pig experiments, (b) CT reconstruction showing the placement of the pressure and aOCT catheters within the porcine airway, and (c) cart-based aOCT/pressure acquisition system.

The pressure acquisition system consists of a ∼1-mm diameter, 130-cm-long catheter pressure transducer (SPR-330A, Millar Inc.) connected to a Pressure Control Unit (PCU-2000, Millar Inc.). The output of the PCU is digitized by the pressure DAQ at 5 kHz using a clock derived by the trigger processor circuit from the 100-kHz laser sweep trigger. The pressure DAQ also digitizes the inspiratory pressure, expiratory pressure, inspiratory flow, and expiratory flow signals from the ventilator. OCT data is processed and displayed in real time using GPU-accelerated software developed by Physical Sciences Inc. along with the pressure and flow signals recorded during the acquisition.

To investigate the system’s suitability for dynamic airway imaging and to validate the measurements made, we performed aOCT and CT sequentially on a paralyzed live pig [Fig. 2(a)]. The animal was placed on the bed of a Siemens Biograph 64 slice CT system (Siemens Medical Solutions USA Inc.). An endotracheal tube with an inflatable cuff was placed in the airway and a dual swivel adapter was connected to it. A SERVO 900C ventilator (Siemens-Elema AB) was connected to the adapter and used to mechanically ventilate the animal. aOCT and pressure catheters were introduced and positioned in the airway through the other port of the swivel adapter under video bronchoscopy, and the bronchoscope was subsequently withdrawn. The pressure catheter was placed near the site of imaging without impeding the OCT field of view [Fig. 2(b)]. An additional stiff polyurethane (PU) sheath was used to steer the OCT catheter and a polyamide sheath attached to the side of the PU sheath was used to house the pressure catheter; these sheaths are seen in Figs. 3(a), 3(b), 4(b), and 4(c). aOCT was performed first, followed by CT; pressure and flow signals were recorded synchronously only during aOCT scans.

**Fig. 3:**
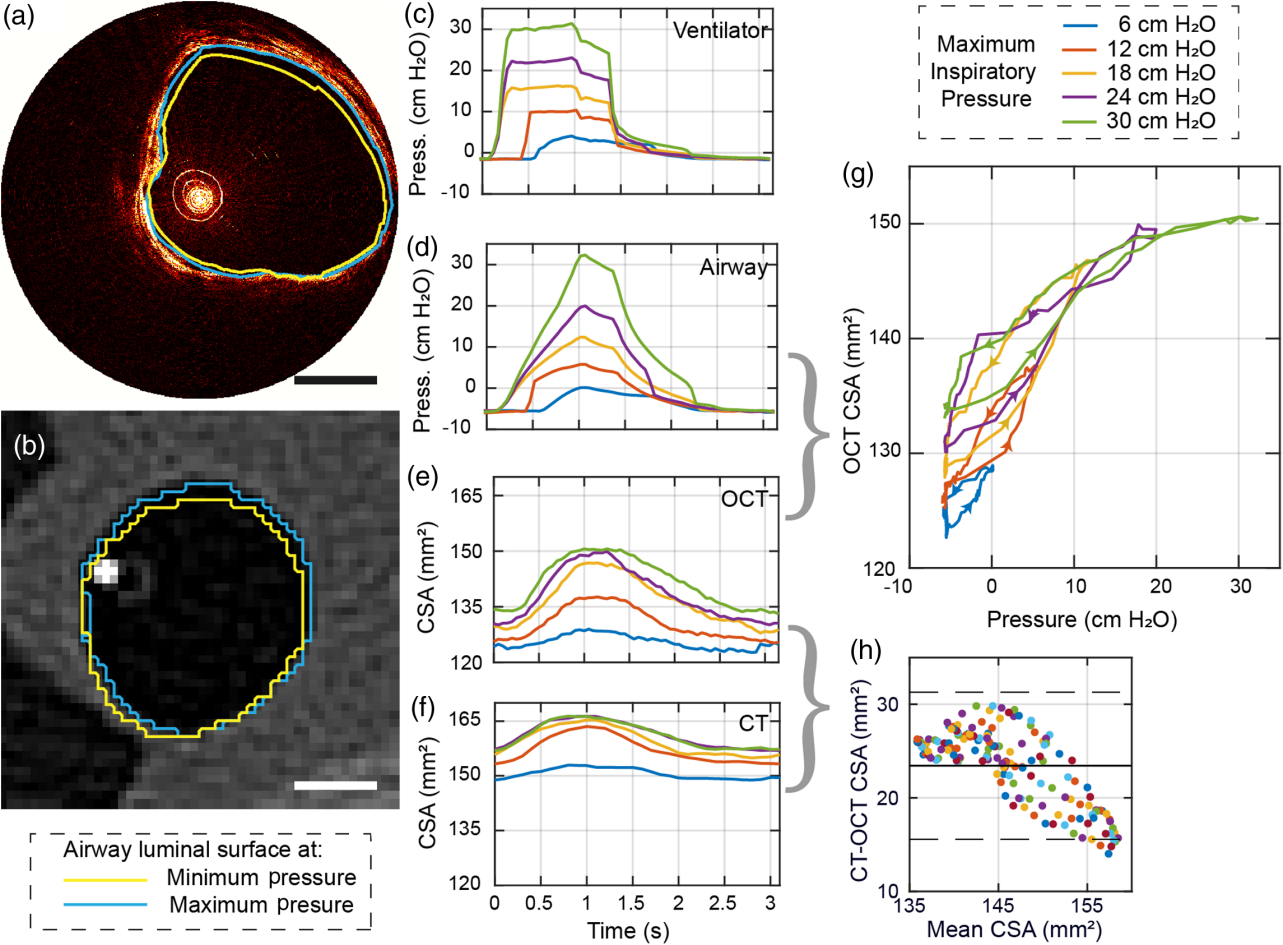
Dynamic airway imaging (a), (b) aOCT and CT images showing the airway under MIP of 12 cm $H_{2}O$ (Video 1); (c) inspiratory pressures measured at the ventilator; (d) intraluminal pressures measured in the airway with the pressure catheter; (e) CSA derived from aOCT scans; (f) CSA derived from CT scans; (g) pressure versus aOCT-derived CSA hysteresis curves; (h) Bland-Altman plot comparing the CT- and aOCT-derived CSA; scale bars=5  mm. (Video 1, MPEG, 1.7 MB [URL: https://doi.org/10.1117/1.JBO.23.10.100501.1].)

The animal was anesthetized with propofol/isoflurane and intubated before experiments began. Subsequently, vecuronium was administered to induce neuromuscular blockade and imaging was performed under mechanical ventilation. The animal was ventilated with weight-appropriate settings prior to and between each acquisition, and vital signs were monitored throughout the experiment by veterinary staff. The experimental protocols used were approved by the Institutional Animal Care and Use Committee at the University of North Carolina, Chapel Hill.

The intraluminal pressure variation and the resulting airway dynamics measured by OCT and CT over one representative respiratory period are shown in Fig. 3. The measurements were performed under pressure-controlled ventilation with maximum inspiratory pressure (MIP) set to values between 6- and 30-cm $H_{2}O$ and a respiratory rate of ∼20 breaths per minute. aOCT was performed at a fixed location in the trachea, above the tracheal bronchus, at a 20-Hz frame rate, and segmented manually to determine the cross-sectional area (CSA) in each frame. Cine CT volumes were acquired over the region around the OCT catheter tip with an in-plane resolution of ∼0.5  mm, slice spacing of 0.6 mm, and frame rate of 10 Hz. CSA was calculated for CT axial images that contained the OCT catheter tip using Mimics (Materialise NV). The CSA results from aOCT and CT scans were synchronized by aligning the minimum CSA with the minimum catheter pressure. Figures 3(a) and 3(b) depict example aOCT and CT images with the airway lumen contours at the extrema (max and min CSA at MIP of 12-cm $H_{2}O$) superimposed (Video 1). The higher resolution of aOCT provides smoother airway lumen contours in comparison with CT. Note that while the aOCT probe position and the CT slice location are anchored, the airway deforms in both the radial as well as the longitudinal directions during the respiratory cycle and is, therefore, a potential confounding factor for both CSA measurements. The overall airway shape also appears different in aOCT compared with CT due to the nonuniform rotation distortion (NURD) caused by variable friction between the rotating catheter and the sheath.

Figure 3(c) shows the inspiratory pressures measured at the ventilator, while Fig. 3(d) shows the pressures measured by the pressure catheter at the aOCT imaging site in the trachea. Although the difference in maximum and minimum pressures measured in the airway is similar to that at the ventilator, the shape of pressure variation within the airway is considerably different from the applied pressure, highlighting the need for intraluminal pressure measurements. Figures 3(e) and 3(f) show the airway CSA variation during a respiratory cycle derived from aOCT and CT images. When plotting the aOCT-derived CSA against intraluminal pressure during inspiration and expiration, we can observe hysteresis in the curves [Fig. 3(g)] that suggest the viscoelastic nature of airway tissue; the arrows on the curves indicate the direction of time. Interestingly, these hysteresis curves resemble pressure–volume curves obtained during pulmonary function testing.^9^ Finally, CSA measured by OCT and CT was compared using a Bland–Altman plot [Fig. 3(h)]; the bias of the measurements was $23\;{\mathrm{mm}}^{2}$ (solid line) with OCT reporting a smaller CSA than CT. The limits of agreement (bias±2SD) were from 15.6 to $31.3\;{\mathrm{mm}}^{2}$ (dashed line). The aggregate difference between all measurements was 15%±3% (mean±SD).

Figure 4 compares the results obtained from aOCT and CT for volumetric imaging across several airway generations. The aOCT scan was performed while the catheter was being rotated at 20 Hz and pulled back at 6  mm/s to obtain a helical scan pattern over a total scan length of ∼100  mm (scan duration 16.7 s). The CT scan was acquired subsequently with only the pressure catheter in the airway, with an in-plane resolution of ∼0.4 mm and a slice thickness of 0.6 mm. The airway was kept at a fixed positive pressure of 12-cm $H_{2}O$ for the duration of the scans. The three-dimensional (3-D) lumen volumes were computed using Mimics. The aOCT-derived volume [Fig. 4(a)] compares favorably with the CT-derived volume [Fig. 4(e)] and is able to capture the location and the orientation of airway branches (numbered arrows on the figures) as well as airway landmarks, like the tracheal bronchus and the tracheal carina, accurately. The OCT polar images that correspond to the positions indicated by the colored lines in Fig. 4(a) are shown in Figs. 4(b)–4(d) and the CT axial images at approximately similar locations are shown in Figs. 4(f)–4(h). It can be seen that the airway shapes and sizes depicted by the two modalities are in agreement with one another, except when the features lie outside the line of sight of the aOCT light beam [Fig. 4(c)] or when the airway shapes are distorted by NURD [Figs. 4(b) and 4(d)]. The region above the tracheal carina is shown clipped on the OCT volume [Fig. 4(a), region above the green arrow] as a portion of the airway surface was outside the imaging range.

**Fig. 4:**
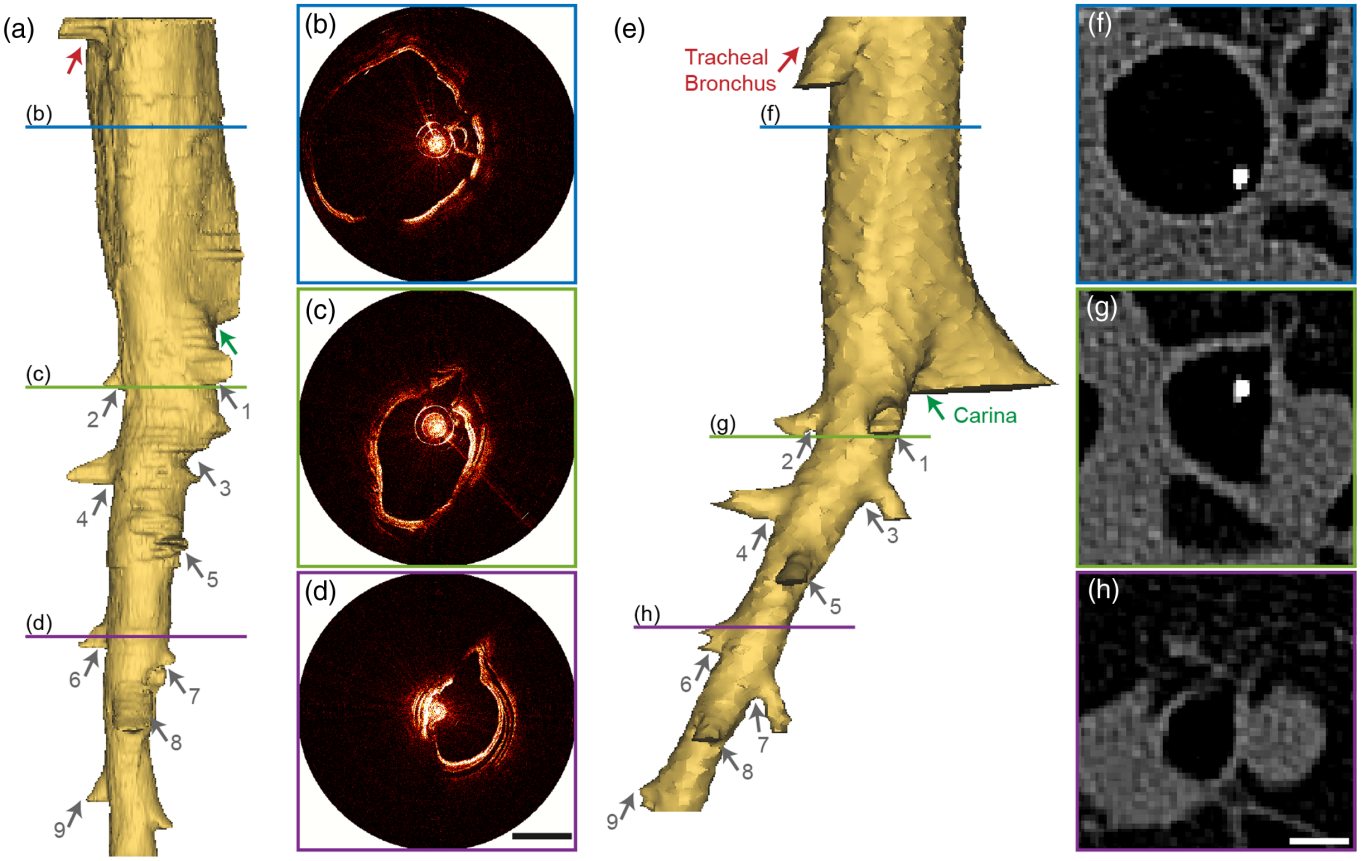
Volumetric data from aOCT scans compared with CT results; (a) 3-D reconstruction from a ∼10-cm-long pullback aOCT scan; (b)–(d) aOCT polar images at the sites indicated by the colored bars on (a); (e) CT derived 3-D volume; (f)–(h) axial CT images corresponding to (b)–(d); labeled arrows on the 3-D volumes indicate corresponding features; scale bars=5  mm.

In conclusion, we have built, characterized, and verified the operation an endoscopic, swept-source aOCT system with synchronized intraluminal pressures that is suitable for anatomical airway imaging. The system has been shown to be capable of accurately quantifying dynamic airway dimensions relative to CT. Using intraluminal pressure changes in the airway during respiration, we have observed hysteresis curves for the porcine trachea *in vivo*; these measurements could be extended to the lower airways to assess the variation in response across airway generations.^6^ These results illustrate the viscoelastic nature of the airway tissue and underscore the benefits of measuring pressures at the site of the imaging. We have also presented a validation protocol that allows dynamic aOCT of airways under varying respiratory conditions to be validated against CT acquired under identical conditions during the same exam. Using this protocol, we anticipate that it will be possible to determine regional differences in airway viscoelastic properties and model dynamic airway collapse in patients suffering from airway obstruction.
